# Switching Adaptability in Human-Inspired Sidesteps: A Minimal Model

**DOI:** 10.3389/fnhum.2017.00298

**Published:** 2017-06-07

**Authors:** Keisuke Fujii, Yuki Yoshihara, Hiroko Tanabe, Yuji Yamamoto

**Affiliations:** ^1^Structured Learning Team, Center for Advanced Intelligence Project, Institute of Physical and Chemical Research (RIKEN)Suita, Japan; ^2^Intelligence Mobility Group, Institutes of Innovation for Future Society, Nagoya UniversityNagoya, Japan; ^3^Graduate School of Arts and Sciences, University of TokyoTokyo, Japan; ^4^Research Center of Health Physical Fitness and Sports, Nagoya UniversityNagoya, Japan

**Keywords:** sensory-motor system, multi-link system, closed-loop system, autonomous distributed control, flexible bipedal locomotion

## Abstract

Humans can adapt to abruptly changing situations by coordinating redundant components, even in bipedality. Conventional adaptability has been reproduced by various computational approaches, such as optimal control, neural oscillator, and reinforcement learning; however, the adaptability in bipedal locomotion necessary for biological and social activities, such as unpredicted direction change in chase-and-escape, is unknown due to the dynamically unstable multi-link closed-loop system. Here we propose a switching adaptation model for performing bipedal locomotion by improving autonomous distributed control, where autonomous actuators interact without central control and switch the roles for propulsion, balancing, and leg swing. Our switching mobility model achieved direction change at any time using only three actuators, although it showed higher motor costs than comparable models without direction change. Our method of evaluating such adaptation at any time should be utilized as a prerequisite for understanding universal motor control. The proposed algorithm may simply explain and predict the adaptation mechanism in human bipedality to coordinate the actuator functions within and between limbs.

## Introduction

We can adaptively operate our bipedal body by cooperating with others in an emergency (Hutchins, 1995; Fujii et al., 2016) and sometimes competing with others (Yamamoto et al., 2013; Fujii et al., 2015b). Current technology can succeed in reproducing such real-time adaptation in video game tasks (Mnih et al., 2015) and overcoming unpredicted deficits (Yoshihara et al., 2007; Cully et al., 2015), although such adaptation is limited to a certain part of the agent's body. However, with regard to bipedal locomotion, which is more dynamically unstable than that of more than four-legged species (Golubitsky et al., 1999), researchers have not paid attention to the adaptive movements when motor commands change suddenly in response to a change in the situation, such as chase-and-escape behavior (Kamimura and Ohira, 2010; Fujii et al., 2015b), which have been acquired in over the course of evolution as biological (Carvalho et al., 2012) and social (Helbing et al., 2000) features essential for life activities. For example, it is considered that a sudden intentional direction change opposite to the original direction, such as in interpersonal sports (Fujii et al., 2015b), is quite difficult to achieve and thus has been ignored in the fields of robotic engineering (Koolen et al., 2016; Kuindersma et al., 2016) and computational neuroscience (Taga et al., 1991; Song and Geyer, 2015), with the focus primarily being placed on bipedal adaptation to external disturbances. Here, we refer to this as switching adaptation in bipedal locomotion because both motor commands (i.e., situation or task) and motor system requirements will switch in this case.

Although most previous studies on human motor control were based on the optimal control theory (Todorov and Jordan, 2002; Scott, 2004), which is considered to be physiologically related to the cerebellum (Shadmehr and Krakauer, 2008), this theory cannot necessarily apply to universal motor control. The theory focuses on optimizing the system based on the centralized invariant cost functions, such as the deviation of target trajectory (Uno et al., 1989) or motor cost as muscle activity (Anderson and Pandy, 2003), such as in arm movement. However, an unstable multi-link closed-loop system with large inertia and a narrow base of support in abruptly changing situations, such as switching adaptation in bipedal locomotion, is difficult to control optimally. This is because it cannot determine the optimal target trajectory due to the large control component with physiological constraints (e.g., joints and muscles), the observation component with cognitive constraints (e.g., ground and opponent) and the context (e.g., the predicted optimal strategy could be defeated by the opponent's counter-attack; Fujii et al., 2015b). Thus, switching adaptation in bipedal locomotion, which is difficult to control even in current robotics (Koolen et al., 2016; Kuindersma et al., 2016), is an excellent example to shed more light on the mystery of universal motor control.

Human bipedality, which is considered to be the result of adaptations to environmental variabilities (Carvalho et al., 2012), is one of the controversial problems to control. While the efficiency of bipedal locomotion in the optimal control theory (Srinivasan and Ruina, 2006) was explained by the dynamics only in the ground phase, neural oscillator control (Taga et al., 1991) that is physiologically located in the spinal central pattern generator (Grillner, 1985; Dimitrijevic et al., 1998) can reproduce the whole of aperiodic adaptive bipedal locomotion in a self-organized manner rather than explicitly calculating the target trajectory or joint torques. However, the oscillator system is considered to be limited in cyclic movement with adaptation only to external disturbances (Thelen et al., 1987; Taga et al., 1991). For example, active adaptation to a changing situation will result in excessive deviation from the aperiodic locomotion generated by the oscillator (e.g., in the opposite direction) because the motor command itself changes drastically. In recent years, using a physiological reflex model, the diversity of walking including a direction change of 50° was reproduced (Song and Geyer, 2015), but in situations such as escape or pursuit, robustly faster direction change at any time (Fujii et al., 2015a) is needed. Furthermore, it is unknown which factors make such adaptive bipedal locomotion difficult because previous locomotion models (Taga et al., 1991; Song and Geyer, 2015) including multiple neural oscillators, peripheral reflexes and multi-link body dynamics were implemented in a complicated manner, whereas as far as the passive walk, the previous model simply accomplished it (McGeer, 1990). Therefore, as a prerequisite for such adaptability, it is important to examine a minimal control model that achieves direction change at any time in the opposite direction with a small number of components and a simple algorithm, and to establish a methodology for evaluating it.

Distributed autonomous control, in which autonomous components implicitly function as a whole by interacting with each other without central control, such as in multi-agent (Couzin et al., 2002) or multi-link (Watanabe et al., 2012) biological systems, is applicable to real-time adaptation to the rapid impairment of components (Yoshihara et al., 2007). This control system is biologically plausible than explicit simulation because the system can perform self-modeling (Bongard et al., 2006) to adapt to the situation beyond its framework. The differences and advantages of the distributed autonomous control compared with the neural oscillator control are that the local components autonomously set the local target and have flexibility in the rule-based interaction among components. Among the autonomous system, self-repairing robots (Bongard et al., 2006; Cully et al., 2015) are remarkable, but the switching adaptation task in this study requires more improvisational adaptation (e.g., within 1 s). The mobility control (Yoshihara et al., 2007) based on the design of autonomous systems, in which an autonomous mobile component moves prior to an immobile component, can execute arm reaching movement when confronting a real-time deficit of the component with improvisational adaptation. We thus assumed that mobility control can be a key factor in the switching adaptability with a minimal algorithm due to the real-time adaptability without the explicit control of the components. However, in bipedal locomotion, in addition to the control of the center of mass in locomotion (equal to endpoint control in arm movement), balance and leg swing control are necessary and often conflict, so not only the operation of equivalent rules for each component but also the switching of rules according to the situation should be important.

In this paper, we adopted switching autonomous system, which extended (i.e., incomplete) distributed autonomous control scheme, because the current task can be accomplished by solving multiple conflicting functions. For example, it would be more advantageous for multiple actuators to switch roles to maintain balance by the leading leg and to move the center of mass by the trailing leg (Yamashita et al., 2013). In neurophysiology, this mechanism may be related to postural control in the reticulospinal tracts found in cats (Mori et al., 1998), but its mechanisms of interaction and switching the function of actuators (i.e., muscles) have remained unknown. We therefore implemented an adaptive bipedal model into role-switching for propulsion, balance, and leg swing control with switching mobility control. The objective of this study is to propose a new control algorithm and evaluation methodology of a switching adaptive model for performing bipedal locomotion as a prerequisite for universal motor control.

## Materials and methods

### Model overview

**Figure 1:**
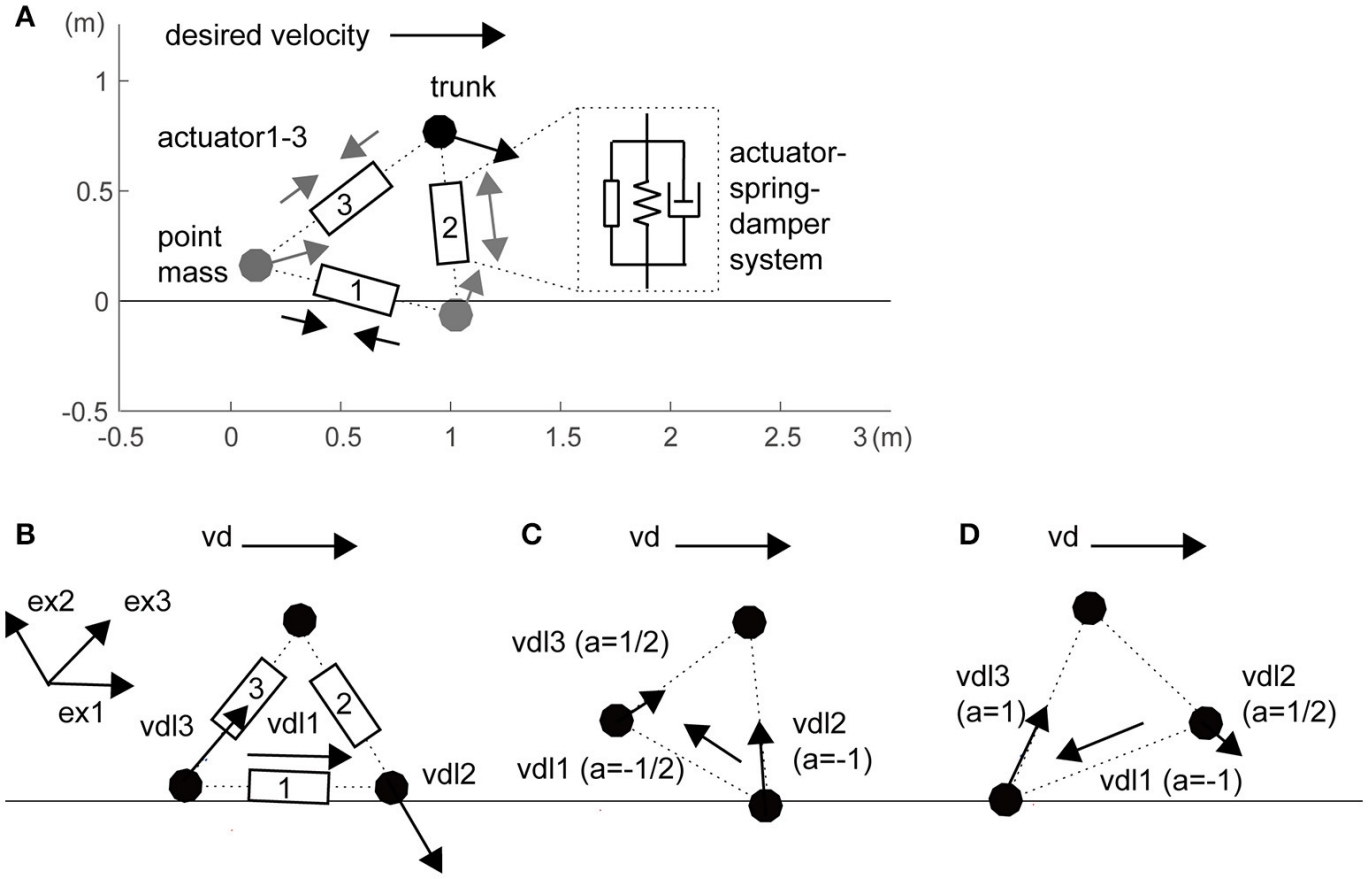
Three-mass locomotion model. **(A)** The three masses (trunk and feet) were linked with three actuators, springs, and dampers that represent the hips (actuator 1) and legs (actuators 2 and 3). For simplicity of spatially symmetrical configuration in the three actuators, inter-leg actuator 1 was modeled as a hip abductor and adductor muscles to swing the legs. These segments were stretchable, but if a leg exceeded a certain length, we increased the elasticity (Table S1). The horizontal desired velocity is given as a single global command from the experimenter. Unidirectional arrows at the three masses and bidirectional arrows at the three actuators illustrate local desired velocities of autonomous actuators and actuator forces, respectively. **(B)** Local desired velocity without balancing (the same as in a previous study; Yoshihara et al., 2007). For propulsion rightward, the inter-leg (actuator 1) and trailing leg (actuator 3) are designed to be lengthened and the leading leg (actuator 2) is designed to be shortened. **(C)** During grounding of the right leading leg, with a risk of falling forward, the inter-leg actuator and the leading leg are designed to be lengthened. **(D)** During grounding of only the left trailing leg, with a risk of raising the leading leg too high, the inter-leg actuator is shortened. Details in a coefficient *a* within brackets and unit vector *e*_*xi*_ are given in Section Materials and Methods.

In this study, we constructed a three-mass model as a toy model (i.e., a minimally redundant model) of a sidestep locomotor system (Figure 1A). The three masses were linked with three actuators, springs, and dampers that represent the legs and inter-leg (*i* = 1–3: inter-leg, right leg and left leg, respectively). For simplicity of the spatially symmetrical configuration of three actuators, inter-leg actuator 1 was modeled as a hip abductor and adductor muscles to swing the legs. Passive parameters are partly based on the human-like model in a previous study (Taga et al., 1991), as shown in Table S1. In this model, the segments were stretchable, but if a leg exceeded a certain length (1.1 times its initial length), we increased the elastic coefficients (Table S1). We also increased the leg elasticity in the foot contact phase compared with that in the flight phase (Table S1).

The model can perform 2D lateral locomotion by sending appropriate commands to the actuators (Figure 1A) according to the following equation of motion:

(1) $$m\overset{..}{x}=\sum_{i}^{3}F_{ai}+mg+F_{passive}$$

where *x* is a position vector of the three mass points, *F*_*ai*_ is an active force vector generated from the three actuators, ***g*** is a gravity acceleration vector, and *F*_*passive*_ is a passive force vector including viscoelasticity of the leg, its extension limit, and auxiliary action in the trunk (Table S1). The last auxiliary viscoelasticity prevents falling if the horizontal distance between the trunk and either leg is within 0.15 m. This value of 0.15 m is heuristically determined based on the trade-off of falling and propulsion in observation. We improved distributed autonomous control (Figures 1B–D), which is based on the rule that the velocity commands are determined from the instantaneous “mobility” of each actuator in real time. This rule will be given in an autonomous decentralized form, which is explained in the paragraphs below.

### Switching mobility control

For switching mobility control, here we consider the velocity command for actuator *i*. Command using positional information is not appropriate in this study because the calculation of the precise target trajectory is not needed. Velocity sensing and command may be reasonable such as due to the utilization of visual optical flow in a self-driven agent. It is assumed that the sensory (i.e., proprioceptive) information of the system including actuator lengths and angles and these derivative values was used. In this section, we consider the following two steps to construct the model: (i) First, the mobility index was defined as the difference between each local actuator's desired velocity ($v_{di}^{l}$) and the actual velocity *v*_*i*_. (ii) Based on the mobility index, global desired velocity *v*_*d*_ was allocated preferentially to mobile actuators and control input in each actuator was determined.

In the first step, actuator *i* divides *v*_*d*_ into two components: a local vector $v_{di}^{l}$ and a residual vector $v_{di}^{r}$. The former is the component of *v*_*d*_ that actuator *i* could generate through its own stretching and shortening and the latter is the component that actuator *i* is incapable of generating in the current leg posture (Figure 1B):

(2) $$v_{di}^{l}=a_{i}\;(e_{xi}\cdot v_{d})\;e_{xi}$$

(3) $$v_{di}^{r}=v_{d}-v_{di}^{l}$$

***e***_*xi*_ is a unit vector to produce the force in actuator *i*. ***e***_*xi*_ in the inter-leg is defined as the unit vector from the trailing leg to the leading leg. Switching coefficient *a*_*i*_ is basically 1 for propulsion but switches for balancing and leg swing based on the related segment sensory information (the schematics are shown in Figure S1). A notable difference from a previous robot arm study (Yoshihara et al., 2007) is that the local desired velocity is modified by coefficient *a*_*i*_ in the situations because of a temporal constraint to apply the force to the ground. When *i* is 2 or 3 (i.e., right or left leg) in the flight phase (Figure S1 right), *a*_*i*_ was set to 1/2 because the leg cannot apply force to the ground and the contribution of the leg to trunk movement halved (Figure 1C: $v_{d3}^{l}$, Figure 1D: $v_{d2}^{l}$). Additionally, when the trunk approaches the anterior leg within 0.2 m, *a*_*i*_ was set to −1 to prevent falling, regardless of being in the flight or supported phase (Figure 1C: $v_{d2}^{l}$). When the posterior leg in the flight phase extended over its natural length, *a*_*i*_ was also set to −1 to attract the leg to the trunk as the swing for the next step (Figure 1C: $v_{d3}^{l}$). These two corrections reflect the balance and leg swing, those conflict with propulsion, respectively.

When *i* is 1 (i.e., inter-leg: Figure S1 left), *a*_*i*_ depended on the phase of both legs. In the double support phase, *a*_*i*_ was set to 0 because of a lack of contribution to trunk velocity. In the double flight phase, *a*_*i*_ was set to 1/2 in the same manner for both legs. Additionally, when the posterior leg length was over 0.6 times the natural length in the double flight phase or the anterior leg support phase, *a*_*i*_ was set to −1 to attract the posterior leg to the trunk. This value of 0.6 was heuristically determined based on the following observation: if it is too large, the model sometimes cannot perform the leg swing, and if it is too small, it cannot move in the desired direction. Because of the dependence on kinematic sensory information of other segments, this system is not purely autonomous. However, this switching system contributed to achieving the task by resolving the trade-off between propulsion and balance.

For the adaptation under various environmental conditions, the mobility measure *k*_*i*_ must evaluate the instantaneous mobility of each joint appropriately, which requires calculation of kinematic and dynamic mobilities (Yoshihara et al., 2007). The kinematic mobility is the ability of actuator *i* to move the trunk, and represented by the absolute value of the local desired velocity, i.e., geometric state of the actuator. Dynamic mobility is the same ability as determined by the dynamic properties of the actuator, which is represented by the difference between the actual state and the geometric state of the actuator. The mobility measure *k*_*i*_ is defined as the corrected ratio of dynamic mobility to kinematic mobility:

(4) $$k_{i}=\text{exp}[-4(ln2)(∥\;v_{di}^{l}-v_{i}{∥}^{2}+\varepsilon_{1})/(∥\;v_{di}^{l}\;{∥}^{2}+\varepsilon_{2})]$$

where ***v***_*i*_ is the velocity produced at the trunk generated by actuator *i*, and ε_1_ and ε_2_ are small values (ε_1_: 10^−10^, ε_2_: 10^−4^) to avoid dividing by zero. The denominator and numerator are related to the kinematic and dynamic mobility of actuator *i*, respectively. The mobility *k*_*i*_ is supposed to take a value of 0 in an immobile actuator, and 1 in a mobile actuator.

Next, by using *k*_*i*_, $v_{di}^{l}$, and $v_{di}^{r}$ (Equations 2–4), we intended to design a real-time controller that would make the most mobile actuator work dominantly, and make the other actuators work cooperatively in order to satisfy *v*_*d*_. Actuator *i* basically tries to move according to its own local vector $v_{di}^{l}$, and require the other actuators to create its residual vector $v_{di}^{r}$. The required velocity from actuator *j* to actuator *i*, $v_{di}^{cj}$, is defined as a projection of $v_{dj}^{r}$ to ***e***_*xi*_:

(5) $$v_{di}^{cj}=(e_{xi}\cdot v_{dj}^{r})e_{xi}$$

The mobility *k*_*i*_ of each actuator then determines how the actuators interact with each other. We express this as follows:

(6) $${\tilde{v}}_{di}=\prod_{j\;\neq \;i}^{m}(1-k_{j})v_{di}^{l}+\sum_{j\;\neq \;i}^{m}k_{j}v_{di}^{cj}$$

$v_{di}^{\sim }$ is the velocity command for actuator *i*. The first term functions as an inhibitory interaction from actuator *j*, which prevents actuator *i* from moving according to its own local velocity $v_{di}^{l}$. In contrast, the second term functions as an excitatory interaction make actuator *i* work cooperatively and generate the residual velocity of actuator *j*. In this study, we considered the minimal model which fully connected among three components (i.e., the nearest neighbors equal to the full connections). In more biological model, note that the number of the connection will increase and we should examine the connection configuration, such as based on the nearest neighbors. The velocity command, $v_{di}^{\sim }$, is transformed to torque as follows:

(7) $$F_{ai}=G_{i}({\tilde{v}}_{di}-v_{i})$$

where *G*_*i*_ is the proportional gain of actuator *i*. We heuristically set it to 3,000 kg/s to perform the task.

### Simulation and statistics

Initial horizontal and vertical positions of the trunk were set as 0 and 0.92 m, respectively. Initially, three masses were kept motionless in an equilateral triangular posture and double support stance. The time step in the simulation was set to 10^−5^ s. To examine the parameter sensitivity, we ideally should use the human parameter for the verification. Although we used the skeletal parameters based on the human parameters (Taga et al., 1991), the passive joint viscoelasticity should be approximated when we expressed it as a scalar value (Taga et al., 1991), because muscle-tendon complex has many components with various viscoelastic properties. Based on the assumption that we can learn the (sub) optimal parameters in various motor tasks, we approximated the passive joint viscoelasticity with the invariant scalar value. Instead, we examined the following three parameters from as broad a range as possible: the elasticity of legs, the elasticity of ground, and the proportional gain of actuators. We simulated the models to multiply each parameter by 10^−1^, 10^−1/2^, 1, 10^1/2^, and 10. The details and the results were given by Figure S3. In short, in the case of multiplying 10^−1/2^ or 10^1/2^ by the each original parameter, the model had sensitivity enough to accomplish the direction change at any time.

To quantify the switching adaptability performance, the reaching time was calculated as the time interval from the direction change command to the movement at 2 m displacement after the direction change. To compare the switching mobility model with the conventional models, we reproduced the two model simulations in forward walking models (Taga et al., 1991; Song and Geyer, 2015). In the neural oscillator model^13^, we used the cited parameters, motion equations and set the constant input as 6. The time step in the simulation was set to 10^−6^ s. In the reflex control model^15^, we used the freely available MATLAB code, and set the type of model to normal walk and the time step of extraction to 10^−3^ s. Other parameters were set as the defaults, including 1.3 m/s as the initial horizontal moving velocity.

For bivariate correlations, we used Pearson's correlation coefficient. For comparing the reaction time between during two different phases, we used the unpaired *t*-test. Both statistics are described with the corresponding degrees of freedom (denoted by a subscript). For all the statistical calculations, *p* < 0.05 was considered significant. All simulations and statistical analyses were performed using MATLAB 2016a Statistics and Machine Learning Toolbox (The MathWorks, Inc., MA, USA).

## Results

### Bipedal locomotion with switching mobility control

**Figure 2:**
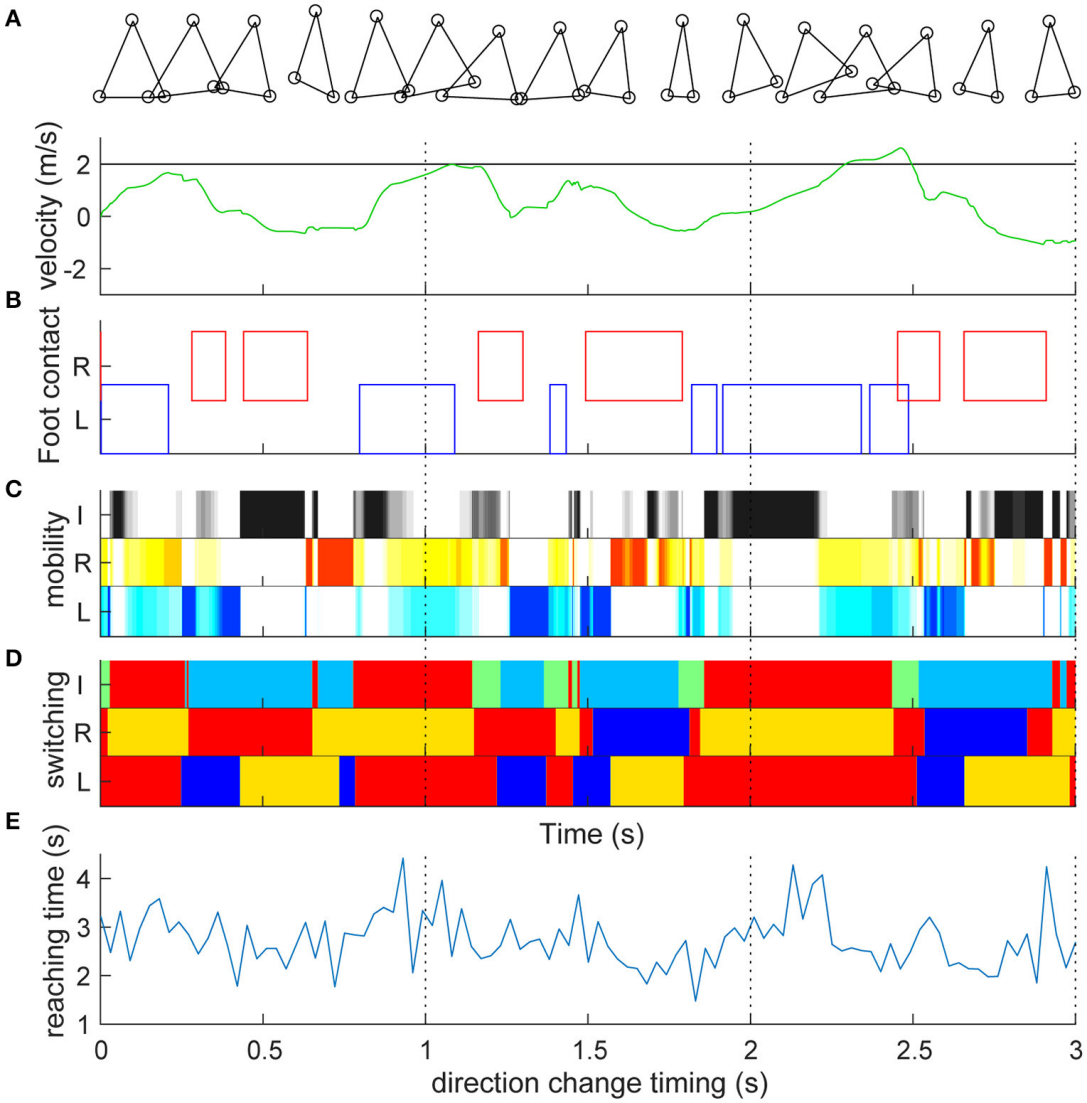
Straight lateral locomotion and a new method of evaluating switching adaptability. **(A)** Time series of the target speed (black line) and actual speed (green line) of the trunk mass (top component). Target speed was set to 2 m/s and actual speed fluctuated. **(B)** Contact with the ground of the right leading foot (red) and the left following foot (blue). The left and right feet were not always grounded alternately. Detailed processes are shown in Video S1. **(C)** Mobility index *k*_*i*_ of inter-leg (I, black, *i* = 1), right (R, red, *i* = 2) and left (L, blue, *i* = 3) legs in the switching mobility control algorithm (a darker color indicates greater mobility). Mobility index of the three actuators alternately increased and decreased to play their roles as determined by the switching coefficients **(D)**. The mobility seemed to increase at the flight phase in both leg actuators and at the grounded phase of either leg in the inter-leg actuator. **(D)** Switching coefficient *a*_*i*_ of actuator *i*'s desired velocity. I, R, and L are the same as in **(C)**. Red (*a*_*i*_ = 1) and blue (*a*_*i*_ = −1) show propulsion and balance during the grounded phase, respectively. Orange (*a*_*i*_ = 1/2) and light blue (*a*_*i*_ = −1/2) show leg swing for propulsion and balance during the flight phase, respectively. Green (*a*_*i*_ = 0) is neutral (i.e., zero velocity command) for the grounded state of either or both legs. The switching coefficients seemed to appropriately switch to propulsion (e.g., grounded in actuators 2 and 3), balance (e.g., grounded in actuators 2 and 1; in flight in actuator 3) and swing (in flight in actuator 2 or 3) separately in each actuator. **(E)** Reaching time (vertical axis) toward 2 m after the direction change command with a 0.03-s interval (horizontal axis). Time series corresponds to the timing of the direction change, which shows high variability (reaching time: 2.734 ± 0.567 s). The reaching time and its variation are the model performance and a new method of evaluating the switching adaptation in bipedal locomotion.

We first set the target speed to 2 m/s and simulated straightforward locomotion without direction change (Video S1). Figure 2A shows the time series of the target and actual velocities of the trunk mass. Similar to actual human bipedal locomotion (Bruijn et al., 2013), the target speed was not always achieved because the acceleration of the body can occur only at the moment when the foot is grounded. The trunk velocity is mostly obtained at the time of contact of the trailing leg (Figures 2A,B) because the leading (right) leg was not designed to overtake the trailing leg in the sidestep (Yamashita et al., 2013). In particular, in this model, alternate grounding of the leading and the trailing feet did not always occur (Figure 2B, Video S1). Thus, the locomotion of our model was neither strictly walking nor running. As is the case for skipping with the repeated same foot contact (Minetti, 1998) and galloping with both the double support and flight phase (Yamashita et al., 2013), in the model, the same foot sometimes repeatedly contacted and showed a double support and flight phase. Our model temporally changed the four gaits characteristics, and thus cannot be categorized. The longer term (60 s) characteristics are shown in Figure S2. The mobility index of the three actuators (Figure 2C) alternately increased and decreased to play their roles as determined by switching coefficients (Figure 2D). Mobility seemed to increase in the flight phase in both leg actuators (2 and 3) and at the phase with either leg grounded in inter-leg actuator 1 (Figure 2C). The switching coefficients seemed to switch appropriately to propulsion (Figure 2D red, e.g., grounded in actuators 2 and 3), balance (blue, e.g., grounded in actuators 2 and 1; in flight in actuator 3), and swing (orange or light blue, in flight in actuator 2 or 3) separately in each actuator (Figure 2D: the algorithm is given in Section Materials and Methods).

### Direction change at various timings

**Figure 3:**
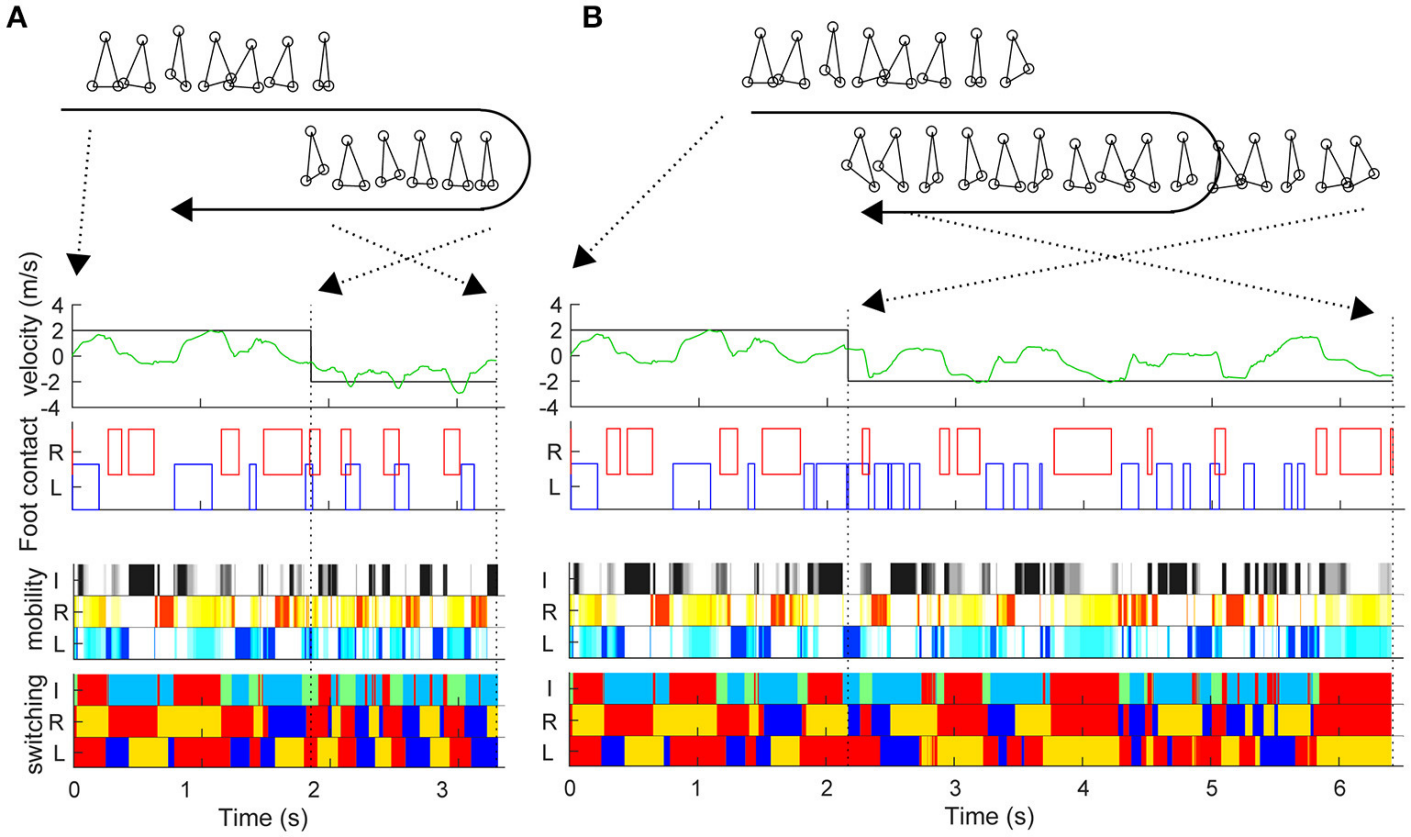
Examples of trials in fast and slow reaching upon direction change. Similar to Figure 2, this figure shows the actual trunk (green) and target (black) velocities, and the left and right foot contacts in the faster direction change after 1.861 s from the start time **(A)** and in the delayed direction change after 2.161 s **(B)**. The upper right and lower right stick pictures are kinematic postures at the direction change command. The moment of the direction change command and at reaching 2 m (simulation end) are indicated by black dotted lines.

During the sidestep in Figure 2, we switched the target speed to −2 m/s at various timings and moved the trunk to 2 m in the opposite direction from that moment. The vertical axis in Figure 2E shows the reaching time toward 2 m after the direction change command with a 0.03-s interval (horizontal axis). First, the switching mobility model achieved direction change at any time using only the three actuators. The reaching time and its variation are the model performance (mean and standard deviation of reaching time: 2.734 ± 0.567 s) and a new evaluation method of the switching adaptation in bipedal locomotion. The results showed the reaching time increased during the trailing leg stance compared with the other timings (2.940 ± 0.644 s vs. 2.604 ± 0.474 s, *t*_99_ = 3.01, *p* = 0.0033). Figure 3 shows examples of the faster direction change (reaching time: 1.478 s) after 1.861 s from the start of the simulation (Figure 3B, Video S2) and in the delayed direction change (reaching time: 4.280 s) after 2.161 s (Figure 3B, Video S3). The faster trial involved a change in direction to switch the mobility index and the switching coefficient and to include fewer steps in a shorter cycle compared with the slower trial. As a coarse grained explanation at the direction change timing, the trailing leg stance increased the reaching time because the trailing leg propelled the body (before the direction change) and then will make the body difficult to change the inverse direction.

**Figure 4:**
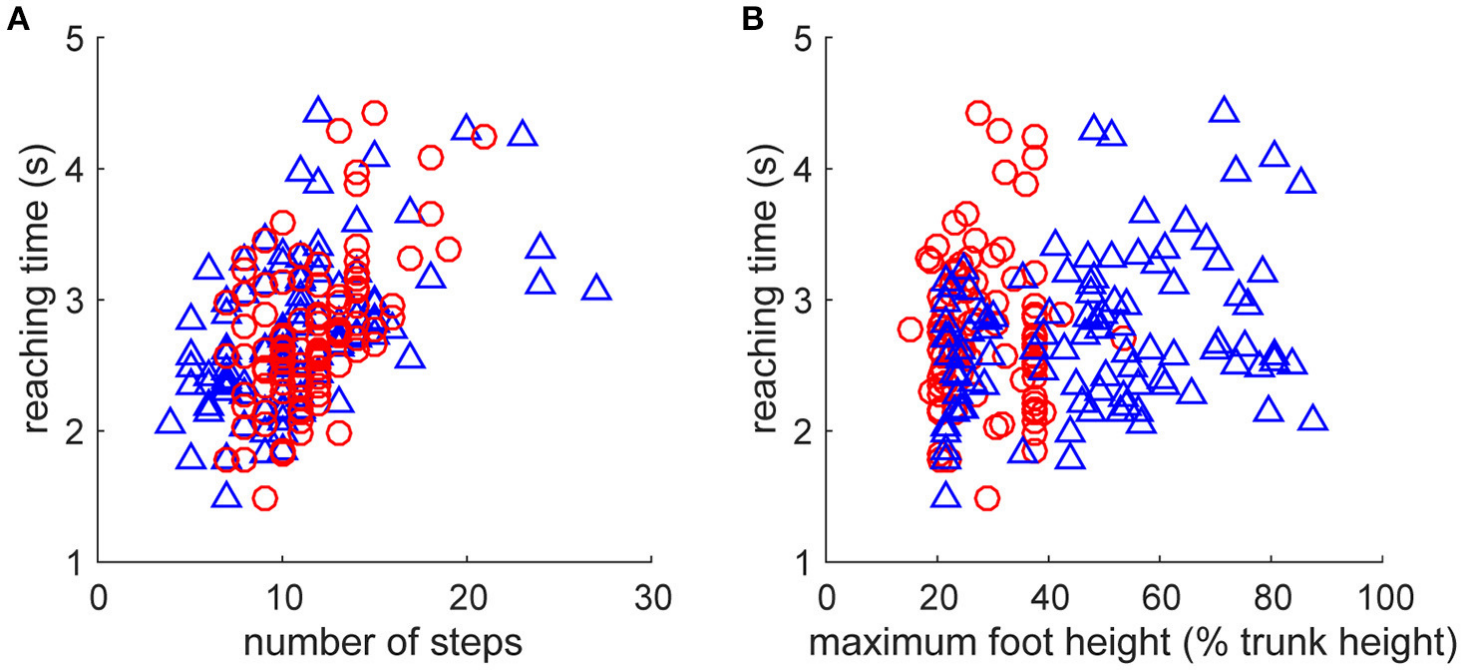
Relationships between kinematic characteristics and performance. Performance was evaluated as the reaction time after the direction change command (Figure 2E). Kinematic characteristics were quantified as **(A)** number of steps and **(B)** maximum vertical foot height of left foot (blue triangle, leading foot) and right foot (red circle, trailing foot) after the direction change. Note that all of the data including foot definition were for after the direction change. **(A)** Reaching time was significantly increased with the number of steps for both leading and trailing feet. **(B)** Reaching time was significantly increased with maximum vertical foot height for the leading foot (blue triangle, left foot), but not that for the trailing foot (red circle, right foot).

As fundamental kinematic characteristics to investigate the fine-grained fluctuation of the reaction time, we examined the relationship of step numbers and foot height with the direction change performance (Figure 4). The reaching time was significantly increased with a greater number of steps for both leading and trailing feet (Figure 4A, leading: *r*_99_ = 0.542, *p* = 4.7 × 10^−9^, trailing: *r*_99_ = 0.509, *p* = 5.4 × 10^−8^). It was also significantly increased with maximum foot height (Figure 4B) for the leading foot (*r*_99_ = 0.283, *p* = 4.1 × 10^−3^), but not that for the trailing foot (*r*_99_ = 0.03, *p* = 0.976). These results suggest that the faster direction change was derived from the movement with less motor cost, estimated by fewer steps and a smaller leading foot height. However, an underlying cause of difference between the trials in the faster and the slower reaching time was difficult to explain directly because the behaviors were interrelated and generated from the closed-loop structure. This may be generated from a subtle dynamic state difference and the subsequent accumulation of integration error in the non-integrable system.

### Comparison with conventional models

**Figure 5:**
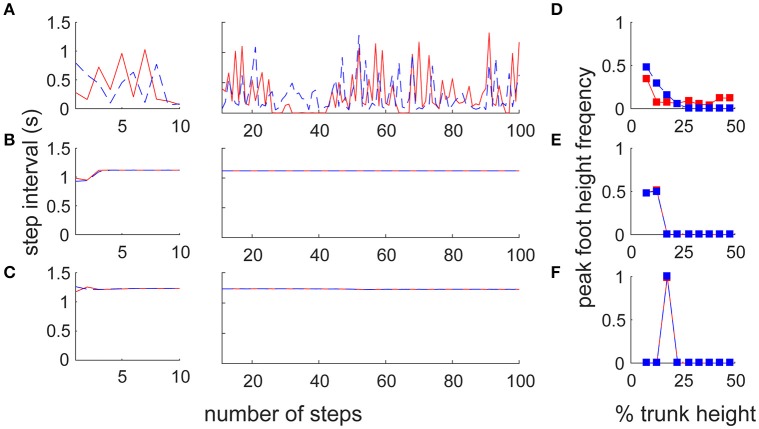
Comparison of step frequency with conventional locomotion models. Time series of step interval of trailing (blue) and leading (red) legs in **(A)** the switching mobility model, **(B)** the neural oscillator model (Taga et al., 1991) and **(C)** the reflex control model (Song and Geyer, 2015). Left and center panels show the first 10 steps magnified and 90 subsequent steps, respectively. Note that the models for comparison were forward locomotion models, but the mean moving velocities were largely different among them (the switching mobility model: 0.575 m/s, neural oscillator model: 1.544 m/s, reflex model: 1.200 m/s). The step interval variability in the switching mobility model was much larger than in the conventional locomotion models. We also included signature leg raising in the switching mobility model and show a histogram of peak foot height frequency in **(D)** the switching mobility model, **(E)** the neural oscillator model and **(F)** the reflex control model. The horizontal axis represents the vertical foot height normalized by the trunk height. Comparable models (especially the computational reflex model; Song and Geyer, 2015) strictly controlled the foot height, but the leading leg in the switching mobility model was sometimes raised higher than in the comparable models.

To reveal the difference in motor output in the different architectures, we reproduced two previous forward walk models with a neural oscillator (Taga et al., 1991) and reflex control (Song and Geyer, 2015; details are given in Section Materials and Methods). Note that because there is no sidestep bipedal model, except for ours, detailed comparisons between the proposed and previous models are impossible (furthermore, these models have parameter sensitivity, so we cannot match the locomotion velocities). We thus focused on the fundamental locomotion characteristics which differed greatly beyond the mere specific parameters of the models. We first compared the amplitude and variance of the inter-step interval for both legs with a large number of steps (90 stable steps from the 11th to the 100th step) to quantify the stability in locomotion (Figures 5A–C). The switching mobility model had much larger variance in step interval, despite the lower horizontal moving velocity (leading leg: 0.298 ± 0.247 s, trailing leg: 0.257 ± 0.190 s at a mean velocity of 0.575 m/s) compared with the forward walking models (neural oscillator model: 1.119 ± 0.001 s at a mean velocity of 1.544 m/s, reflex model: 1.229 ± 0.004 s at 1.200 m/s; in both models, the intervals for the left and right legs were the same). The step interval variance in the switching mobility model was also much larger than the measured human sidestep walking data (Yamashita et al., 2013; ~1.0 ± 0.1 s at 1.3 m/s). In addition, from a visual analysis, in the switching mobility model, sidestepping was performed in a less efficient way with higher foot raising (Figure 5D) or a repeated grounded phase in the same leg (Figure 2C, Video S1) at the expense of switching adaptability. We calculated the peak foot height frequency (Figures 5D–F) and showed that the leading leg was sometimes raised higher than in the comparable models, in which the foot height was strictly controlled, especially in the computational reflex model (Song and Geyer, 2015).

## Discussion

In this study, we constructed a minimal distributed autonomous model achieving bipedal change in direction at any time with only three actuators, but without accurate features representing the whole human body structure, such as a central pattern generator and a lower limb joint, as reproduced in the previous comparable models (Taga et al., 1991; Song and Geyer, 2015), that is, without explicit optimization and cyclic movement in neural oscillators. Simultaneously, we also developed a new method for evaluating the ability to perform robustly faster direction change during bipedal locomotion (Figure 2E). In previous studies of adaptive bipedal locomotion in robotic engineering or neurophysiology, such as when encountering an obstacle in the environment or an external disturbance (Taga et al., 1991; Song and Geyer, 2015; Koolen et al., 2016; Kuindersma et al., 2016), it was not considered whether the bipedal model can achieve direction change at any time, which is not explicitly implemented (i.e., unpredictable for the model). Thus, to discuss this control problem, it is necessary to reconstruct the frameworks from the viewpoints of engineering control and neurophysiology, as explained below. The expansion of the frameworks should be necessary for understanding universal motor control.

From the viewpoint of engineering control, the switching mobility model showed switching adaptability at the expense of efficiency because it is difficult for the bipedal locomotion model to satisfy the criteria of both efficiency and adaptability. Previous research using an inverted pendulum locomotion model (Srinivasan and Ruina, 2006) explained the energy efficiency in various locomotion patterns based on the optimal control theory. With regard to efficiency, the switching mobility model showed highly costly movement in which a period of repeated grounding of the same leg occurred (Figure 3), in contrast to that in the conventional models (Taga et al., 1991; Song and Geyer, 2015), even without a change in direction. Furthermore, at the time of changing direction, it sometimes takes a long time to adapt to the command to change direction (Figure 3B) because of a greater number of steps (Figure 4A) and a higher leading leg raise (Figure 4B). This leg raise was controlled in the neural oscillator model (Taga et al., 1991) and strictly computed in the reflex model (Song and Geyer, 2015) at a lower level than in the switching mobility model without direction change (Figure 5). In adaptive bipedal locomotion, it is difficult to calculate the optimal trajectory, so we confirmed the higher motor cost in the switching mobility model, estimated from the much larger variance in step interval and much higher leg raise, than in the conventional models. This is also because there is no mathematical guarantee of efficient movement in mobility control (Yoshihara et al., 2007), which is different from the explicit optimal control or neural oscillator control in cyclic movement. Instead, switching mobility control in the switching mobility model would have an advantage regarding adaptability, even if the movement switches from a cyclic to a non-cyclic pattern and vice versa and has difficulty in control, such as large instability as in bipedal chase-and-escape in interactive sports (Fujii et al., 2015b,c). We were not able to perform a direct comparison with these control algorithms because there are no models to show the switching adaptability (and performing a sidestep); however, a future model can be compared by the proposed evaluation method in which the model performs direction change at various timings.

Neurophysiologically, our switching mobility control algorithm suggests the presence of reflex-like switching functions of propulsion, balancing, and leg swing within and between limbs to achieve the task. The algorithm does not directly reflect the neural mechanism, but we can consider similarity with human neurophysiology by a process of elimination. The proposed model does not explicitly control the actuator movements like cerebellum (Shadmehr and Krakauer, 2008) and does not directly generate quasiperiodic movements such as using neural oscillators (Taga et al., 1991) physiologically located in spinal central pattern generators (Grillner, 1985; Dimitrijevic et al., 1998). Instead, the previous work suggests that diverse movements can be reproduced by incorporating the multiple reflexes in the spinal feedback circuitry without the central pattern generator (Song and Geyer, 2015). Among these architectures, we can find the similarity between the reflex control and the autonomous distributed control (Yoshihara et al., 2007; Watanabe et al., 2012), which incorporates interaction among components and environments (i.e., feedback of ground reaction force) and can implicitly adapt to a rapid change of command. In other words, the point at which each actuator autonomously sets and executes a target movement according to the situation in the switching mobility model matches that in the physiological reflex mechanism in which the inputs and outputs are locally automated. Our switching distributed autonomous control algorithm provided the relationship among the actuators (i.e., muscles) to switch functions of propulsion, balancing, and leg swing within and between limb levels. In a manner similar to long-latency reflexes, which would possess an internal model of limb dynamics (Kurtzer et al., 2008), the actuator in the switching mobility model rapidly switched functions according to the situation. There is no physiological evidence from the neural circuitry of a response of such inter-limb reflexes to a drastic change of command in the long neural pathway, but we believe that it would be needed for the switching adaptation in bipedal locomotion. The neural mechanism involved would be complicated because of possible involvement with both voluntary and reflex control overlapping in their neural substrates (Kurtzer et al., 2008); however, it may be simply explained by the simple interaction rule of our switching mobility control. This constructive approach can contribute to understanding intelligent motor control including biologically (Carvalho et al., 2012) and socially (Helbing et al., 2000) essential activities, such as escape from enemies, pursuit of prey, and search for food.

However, there are some problems with the above neurophysiological claims. One is that it claims to be based only on the similarity in the architectures without neurophysiological evidences. This is considered as a general problem in finding evidences of long-latency reflexes, which overlapped with voluntary movements in their neural substrates (Kurtzer et al., 2008). Second is validation with real-world human data satisfying both sidestepping (Yamashita et al., 2013) and unpredicted change direction (Fujii et al., 2015b). The latter study showed that the unpredicted competitive situation (i.e., requiring the faster movement) delayed the first step initiation more than 100 ms, suggesting the human can decrease the delay more than the current switching adaptability model. The real human locomotion mechanism includes central pattern generators, peripheral reflexes, and spring-damper system. The spring-damper property, which contributes high speed locomotion according to a quadruped robot study (Kimura et al., 2007), should be further investigated.

Third is the sensitivity of the simulation to the choice of some of the model parameters. Our supplementary results (Figure S3) showed that the switching adaptability model had strong sensitivity to the parameters. We suppose the parameterization may be related with inherent adaptation to the individual musculoskeletal system and might be relatively independent of the motor control adaptation. As a further alternative approach, for example, evolutionary algorithm (Song and Geyer, 2015) and reinforcement learning (Lillicrap et al., 2015) efficiently worked in previous bipedal locomotion studies. These are complementary relationship in terms of inter- and intra-generation progress, respectively. The reflex model (Song and Geyer, 2015) optimized the control parameters with the covariance matrix adaptation evolution strategy (Hansen, 2006). Reinforcement learning enables the acquisition of efficient and adaptive locomotion by trial and error on models, like humans actually do, probably in the basal ganglia (Doya, 2000). However, if learning a case of sudden change through evolutionary algorithm and reinforcement learning (also in learning of humans as an experimental condition; Fujii et al., 2013), there is a possibility that it will not mean “unpredictable sudden change” when performing an adaptive bipedal locomotion (Shinya et al., 2009). In other words, it can be considered that the explicit control rule should be difficult to estimate in principle in these approaches (i.e., human bipedal locomotion can also predictively adapt to the situation; Shinya et al., 2009). The evolutionary algorithm and reinforcement learning will also have the advantage to acquire the efficient movement in our less efficient model. Satisfying the requirements of efficiency (e.g., obtained explicitly by using optimal control and implicitly by evolutionary algorithm or reinforcement learning) and switching adaptability proposed by our study will further contribute to solving the general motor control problem.

## Author contributions

KF conceived the original idea of the model. KF and YYo designed the model. KF, YYo, HT, and YYa analyzed data and wrote the paper.

### Conflict of interest statement

The authors declare that the research was conducted in the absence of any commercial or financial relationships that could be construed as a potential conflict of interest.
