# Supplementary material for: Switching Adaptability in Human-Inspired Sidesteps: A Minimal Model
Source: Front Hum Neurosci. 2017 Jun 7;11:298. doi: 10.3389/fnhum.2017.00298 (PMC5461270; doi:10.3389/fnhum.2017.00298)
Supplement: Supplementary file 1 [file Presentation1.PDF]

Supplementary Material

Switching adaptability in human-inspired sidesteps: A minimal model

Keisuke Fujii*, Yuki Yoshihara, Hiroko Tanabe and Yuji Yamamoto

*** Correspondence:** Keisuke Fujii: keisuke198619@gmail.com

1 Supplementary Figures and Tables

1.1 Supplementary Figures

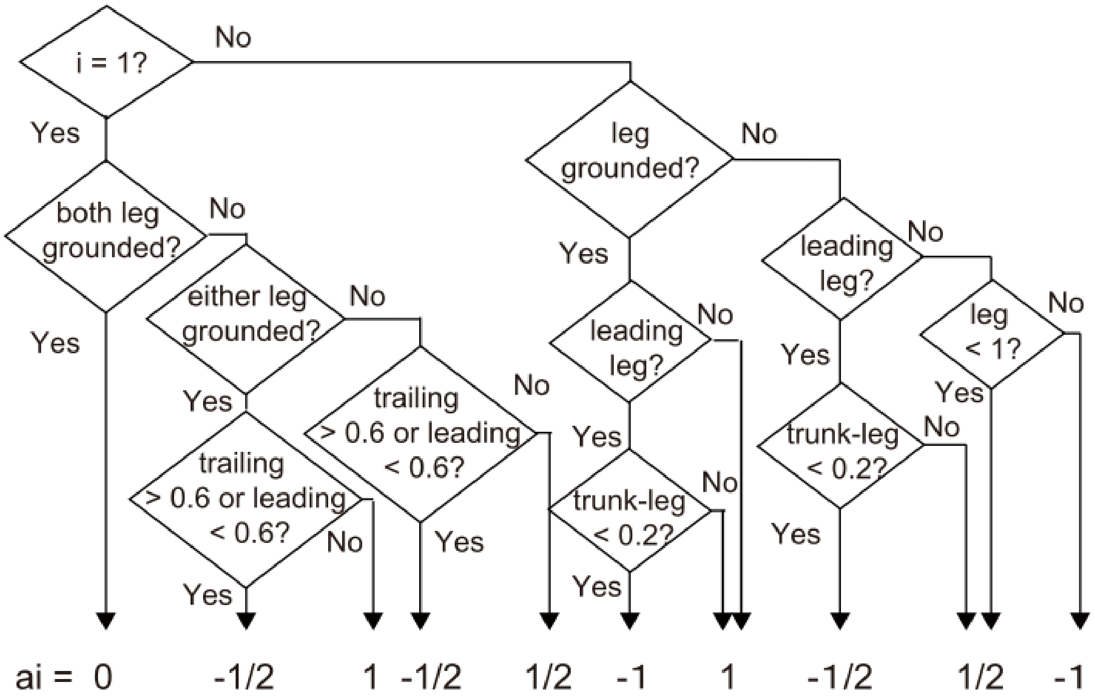

Figure S1. Schematic diagram of determination of switching coefficient. Details are explained in the Methods section.

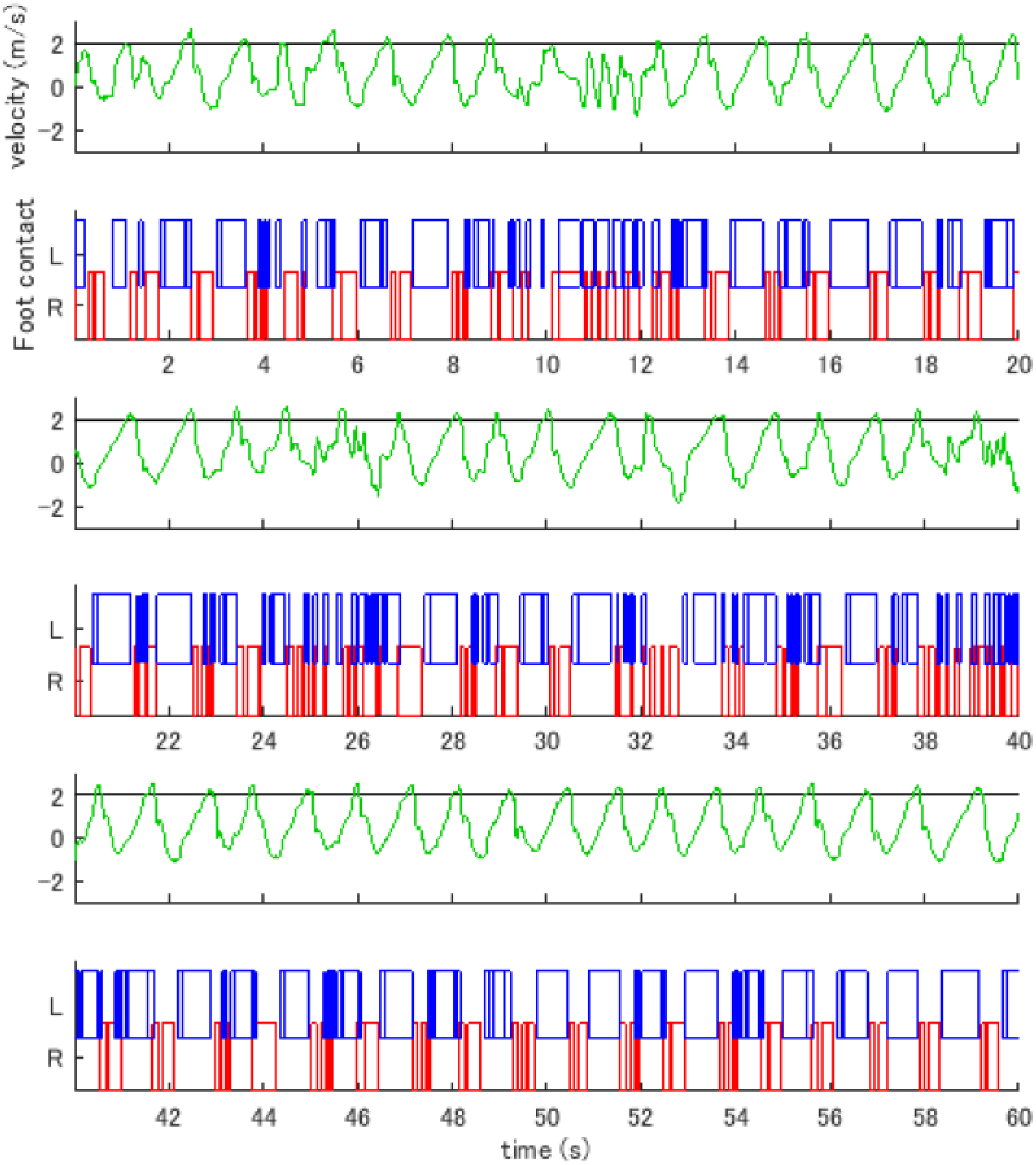

Figure S2. Straight lateral locomotion of the proposed model in 60 s. Configuration and initial condition are the same as Figure 2.

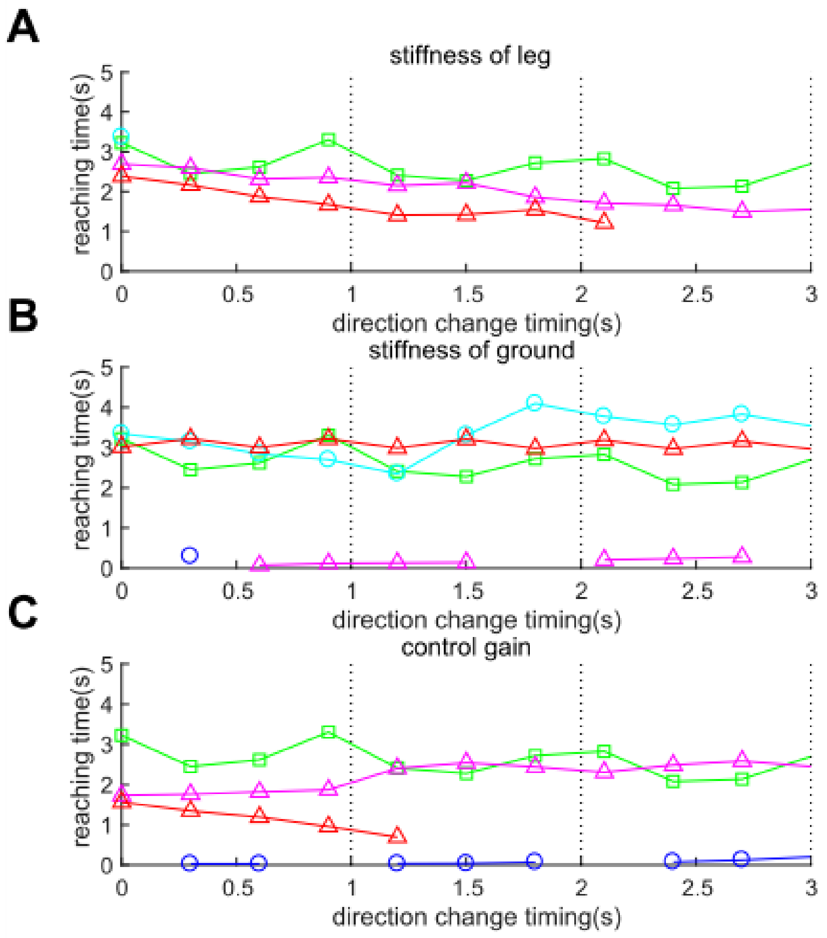

Figure S3. The three parameter sensitivities. (A) The elasticity of legs, (B) the elasticity of ground, and (C) the proportional gain of actuators (Table S1) were examined. We simulated the models to multiply each parameter by 10^{-1}, $10^{-1/2}$, 1, $10^{1/2}$, and 10 (colors were blue, light blue, green, magenta, red, respectively). To investigate coarse-grained performance evaluations, we only investigate 11 time points with a 0.3-s interval (horizontal axis). Performances were defined as the reaching time (vertical axis). If the model cannot reach within the given simulation step (10 s) or fell to the ground, we did not display it on the graph. With respect to (A) the elasticity of legs, the elasticity in both flight phase and for both left and right legs were simultaneously changed. The simulation with only the original parameter and the parameter multiplied by $10^{1/2}$ accomplished the direction change task at any time. Regarding (B) the elasticity of the ground, the simulation with the original parameter and the parameter multiplied by $10^{-1/2}$ and 10 accomplished the direction change task at any time. The simulation with the parameter multiplied by $10^{1/2}$ or 10^{-1} showed unstable results in which it sometimes performed too faster direction change (but the other time it cannot perform the direction change). These parameter changes contributed to not only the stability in the controller but also that in the dynamic simulator itself (including the environments), in which the behavior did not seem to be a natural locomotion. Similarly, with respect to (C) the control gain, there were too faster and strange locomotion with the parameter multiplied by 0.1. The simulation with only the original parameter and the parameter multiplied by $10^{1/2}$ accomplished the direction change task at any time.

1.2 Supplementary Table

Table S1. Simulation parameters.

Parameter	Value
Mass of trunk: m_1	48 kg
Mass of foot: m_2, m_3	11 kg
Length of segment: l_{01}, l_{02}, l_{03}	1 m
Elasticity of leg (flight phase): k_{21}, k_{31}	5000 N/m
Elasticity of leg (support phase): k_{22}, k_{22}	10000 N/m
Viscosity of leg: b_2, b_3	300 Ns/m
Segment extension limit length: l_{lim1}, l_{lim2}, l_{im3}	1.1 m
Elasticity of leg extension limit: k_{lim1}, k_{lim2}, k_{lim3}	100000 N/m
Viscosity of leg extension limit: b_{lim1}, b_{lim2}, b_{lim3}	100 Ns/m
Elasticity of ground: k_g	100000 N/m
Viscosity of ground: b_g	100 Ns/m
Auxiliary elasticity of trunk: k_{at}	5000 N/m
Auxiliary viscosity of trunk: b_{at}	500 Ns/m

2 Supplementary video legends

Video S1. Straight lateral locomotion of the proposed model. Time series. X axis and Y axis in the upper part are lateral and vertical coordinate [m], respectively. The lower part is the time series of the desired and actual velocity (the same as Figure 2). X axis indicates the time [s]. Tr = 0 indicates straight lateral locomotion without direction change.

Video S2. Earlier direction change of the proposed model. Configures are same as Video S1. Time series are the same as Figure 4A. DC is the direction change timing. Tr indicates the number of direction change timing (total 101 trials: 3 s with a 0.03-s time shift). RT shows the reaching time.

Video S3. Delayed direction change of the proposed model. Configures are same as Video S2. Time series are the same as Figure 4B.
